# Dichotic Sentences Test Performance of Adults with communication complaints

**DOI:** 10.1590/2317-1782/20232021301en

**Published:** 2023-08-07

**Authors:** Geise Corrêa Ferreira, Eliane Schochat, Katya Marcondes Freire, Maristela Julio Costa

**Affiliations:** 1 Programa de Pós-graduação em Distúrbios da Comunicação Humana, Universidade Federal de Santa Maria - UFSM - Santa Maria (RS), Brasil.; 2 Departamento de Fisioterapia, Fonoaudiologia e Terapia Ocupacional, Faculdade de Medicina, Universidade de São Paulo - USP - São Paulo (SP), Brasil.; 3 Audicare - São Paulo (SP), Brasil.

**Keywords:** Hearing, Auditory Perception, Hearing Tests, Adult, Medical History Taking

## Abstract

**Purpose:**

To analyze the performance of normal-hearing adults with communication complaints in the Dichotic Sentences Test.

**Methods:**

We selected from the database 15 normal-hearing participants with normal results in the Digits Dichotic Test, aged between 19 and 44 years, right-handed, who reported communicative complaints. The Dichotic Sentences Test was applied using two protocols consisting of four different combinations of lists called sequences 1 and 2, in the following order: training, divided attention step, right and left directed attention steps.

**Results:**

In the first application sequence, the average performance in the divided attention step was 84.7% for the right ear and 60.67% for the left, with statistical difference between ears. The asymmetry between ears varied from -50% to 60%. In the directed attention step, the average performance was 99.33% for the right ear and 98% for the left, with no statistical difference. In the second application sequence, there was a tendency for better results, more pronounced for the left ear, with no statistical significance, with the performance variation and asymmetry between ears remaining high. In the comparison between the sequences, in the divided attention step, it was found that, for the right ear, 40% of the individuals did not vary, 33% performed worse, and 26.7% performed better; for the left ear, 6.6% did not vary, 20% performed worse, and 73.33% performed better. There was result stability in the directed attention step.

**Conclusion:**

The normal-hearing adults with communication complaints presented a heterogeneous profile, especially in the divided attention step, with a marked difference between ears and response variability.

## INTRODUCTION

The sense of hearing is what makes us able to apprehend the physical world of sound vibrations and, from that, form the mental images of the lived experiences^(1)^. In our everyday lives, we are exposed to countless simultaneous auditory stimuli, requiring hearing integrity not only at the peripheral level but also at the central level for effective communication to be established^(2)^.

In the clinical routine, there is an increase in adult individuals who seek audiological assessments for presenting low academic or professional performance, reporting complaints such as difficulties hearing, paying attention, and memorizing, but especially understanding speech, primarily in unfavorable situations ​(with competing verbal messages) However, a large share of such individuals presents auditory thresholds within normality standards, which, in turn, renders the performance of the Central Auditory Processing (CAP) assessment fundamental to investigate the origin of such complaints^(3)^.

CAP is a set of auditory skills responsible for the perception and processing of acoustic information^(4)^. For its assessment, there is a wide range of behavioral tests that evaluate the auditory function and its relationship with communication^(5)^. Among them are the dichotic listening tests, with the use of at least one of such tests in CAP assessment being proposed^(4,6)^.

This type of task may have, as a verbal stimulus, syllables, digits, words, and sentences, and corresponds to the concomitant presentation of two competing stimuli in both ears, enabling the performance of binaural integration and separation tasks with the purpose of investigating the figure-ground skill^(7)^, in addition to providing information on the attention, auditory pathway maturation, executive function, and hemispheric and inter-hemispheric function via the corpus callosum^(8-10)^.

Recently, the Dichotic Sentences Test (DST) was developed^(11)^, aiming to investigate the performance of an individual in competing listening situations through stimuli composed of simple sentences that represent everyday situations.

For being a new test, with characteristics differing from the other dichotic listening tests found in the Brazilian and international literature, measures are necessary to establish parameters in different populations that guide the evaluator in the interpretation of the results, which, thus, justifies the conduction of this research, focused on investigating the test sensitivity. Considering the aspects mentioned above, this study aimed to analyze and compare the performance of normal-hearing adults with communication complaints in the Dichotic Sentences Test in application sequences 1 and 2.

## METHODS

This is an applied, experimental, quantitative-analytical study of retrospective nature conducted by analyzing the database connected to a research project previously approved by the Research Ethics Committee under protocol No. 2.764.720. To compose the study group, the assessments performed from October 2016 to February 2017 of normal-hearing adult patients aged 19 to 44 years were analyzed.

The study group was selected from a database consisting of 101 individuals submitted to a specific anamnesis, basic audiological assessment, to obtain the tone thresholds and middle ear conditions. As for the assessment of the auditory skills of binaural integration and separation, the Dichotic Digit Test was performed in the integration stage, and the dichotic test of sentences, sequences 1 and 2, in the stages of divided and directed attention to the right and to the left, whose stimuli were presented at a level of 50 dB SL in relation to the tritone average (frequency of 500, 1000 and 2000 Hz).

To select the sample, we used the following eligibility criteria: having normal-hearing, considering audibility thresholds less than or equal to 25 dB HL^(12)^ with Dichotic Digits Test (DDT) performance greater than or equal to 95%^(1)^ for both ears in the binaural integration step; being right-handed; having at least a complete high school education; having referred in the anamnesis one or more complaints related to the difficulty of understanding speech in unfavorable listening situations, such as in reverberant environments or in the presence of competitive noise, as well as presenting a complaint of tinnitus and aspects related to attention and memory, emphasizing that these were pointed out by them as factors that could be associated with the main complaint mentioned.

We excluded individuals who presented earwax excess, possible conductive aspects, and evident or self-reported neurological and/or verbal fluency alterations. Hence, the sample group was composed of fifteen individuals, with six male and nine female and an average age of 28.7 years.

The measurements with the Sentences Dichotic Test were obtained by applying two protocols composed of four different combinations of lists called Sequences 1 and 2, according to the following order: training, divided attention step, right and left directed attention step^(11)^. All participants were instructed on how to present the stimuli and the response (verbal repetition) required for each step of the assessments.

The second application sequence was performed with the purpose of investigating the repeatability of the results obtained in the first application, given that it is a new instrument with verbal stimulus and application characteristics different from all dichotic tests that exist in Portuguese and, for this reason, there is no test that may be considered the gold standard for analyzing comparison and sensitivity.

Regarding the DST performance analysis, the score determined was 10% for each sentence, considering that each application list of both sequences is formed by a set of ten pairs of sentences, thus totalizing 100% per ear for each step. To assess the responses obtained in the different test steps, the complete repetition of the entire presented sentences was considered correct^(11)^.

To analyze the differences in the scores obtained between the ears, it was considered that a negative score meant that it was better for the left ear, a zero score, that there was no difference between the ears, and a positive score, that it was better for the right ear.

In turn, when the differences in the scores obtained in the first and second application sequences were analyzed, they were considered according to the ear side using the following criteria: obtaining a negative score meant that the performance of the respective ear was better in the second sequence than in the first; a score of zero meant that the same score was obtained in both sequences; a positive score meant that the first sequence had a better score than the second.

The assessments were carried out by a single evaluator in an acoustically treated room with a Grason-Stadler GSI-61 audiometer and TDH 50 in-ear headphones. The DDTs and DSTs were presented digitally recorded in WAV format through a tablet coupled to the audiometer.

The data were analyzed statistically using the SPSS program. The Shapiro Wilk test was applied using the 5% level as a significance determination criterion to verify the normality of the variables. The non-parametric Wilcoxon test was used to investigate the performance of the individuals in the DST and the different applications of the instrument since the normality hypothesis was rejected. The analyses that presented p ≤ 0.05 were deemed statistically significant.

## RESULTS

Table 1 shows the absolute performance values in the divided and directed attention steps of the DST of adults with communication complaints in Table 1.

**Table 1:** Analysis of the performance of normal-hearing adults with communication complaints in the Dichotic Sentences Test in the divided and directed attention steps in the first application sequence

	**DIVIDED ATTENTION**			**DIRECTED ATTENTION**		
RIGHT EAR	LEFT EAR	P-VALUE	RIGHT EAR	LEFT EAR	P-VALUE
Average (SD)	84.67 (16.85)	60.67 (19.07)	0.020*	99.33 (2.58)	98 (4.14)	0.317
IR	90 (70-100)	60 (50-60.0)		100 (100-100)	100 (100-100)	
MIN-MAX	40-100	30-100		90-100	90-100	

* Wilcoxon test, significance of p ≤ 0.05

**Caption:** SD = Standard Deviation; IR = Interquartile Range; MIN-MAX = Minimum-Maximum

In Table 2, one may observe the differences in the performance obtained between the right and left ears in the divided attention step of the first application sequence.

**Table 2:** Differences between the scores obtained between the right and left ears in the first application sequence in the divided attention step of the Dichotic Sentences Test (n = 15)

Difference (RE-LE)	1st Application Sequence n (%)
-50	1 (6.66)
-30	1 (6.66)
-10	1 (6.66)
0	0 (0)
10	2 (13.33)
30	1 (6.66)
40	6 (40.0)
50	2 (13.33)
60	1 (6.66)

**Caption:** n = Number of Individuals; RE = Right Ear; LE = Left Ear

The comparison of the performance of the adults with communicative complaints in the divided and directed attention steps between DST Application Sequences 1 and 2 may be observed in Table 3.

**Table 3:** Comparison between the performances obtained by adults with communication complaints in the Dichotic Sentences Test in the divided and directed attention steps in the first and second application sequences

	1st Application Sequence			2nd Application Sequence			
STEPS	AVERAGE(SD)	IR	MIN-MAX	AVERAGE(SD)	IR	MIN-MAX	P-VALUE
DIV. ATT. RE	84.67 (16.85)	90 (70-100)	40-100	86 (13.52)	90(70-100)	60-100	0.809
DIV. ATT. LE	60.67 (19.07)	60 (50-60)	30-100	70 (15.11)	70(60-80)	50-100	0.055
DIR. ATT. RE	99.33 (2.58)	100 (100-100)	90-100	99.33 (2.58)	100(100 100)	90-100	1.00
DIR. ATT. LE	98 (4.14)	100 (100-100)	90-100	96.67 (6.17)	100 (90-100)	80-100	0.480

Wilcoxon test

**Caption:** SD = Standard Deviation; IR = Interquartile Range; MIN-MAX = Minimum-Maximum; DIV. ATT. = Divided Attention; DIR. ATT. = Directed Attention; RE = Right Ear; LE = Left Ear

Table 4 presents the performance variations between the right and left ears obtained with the second DST application sequence.

**Table 4:** Differences between the scores obtained between the right and left ears in the second application sequence in the divided attention step of the Dichotic Sentences Test (n = 15)

Difference (RE-LE)	2nd Application Sequence n (%)
-30	2 (13.33)
-20	1 (6.66)
0	2 (13.33)
20	3 (20.0)
30	3 (20.0)
40	3 (20.0)
50	1 (6.66)

**Caption:** n = Number of Individuals; RE = Right Ear; LE = Left Ear

**Figure 1:**
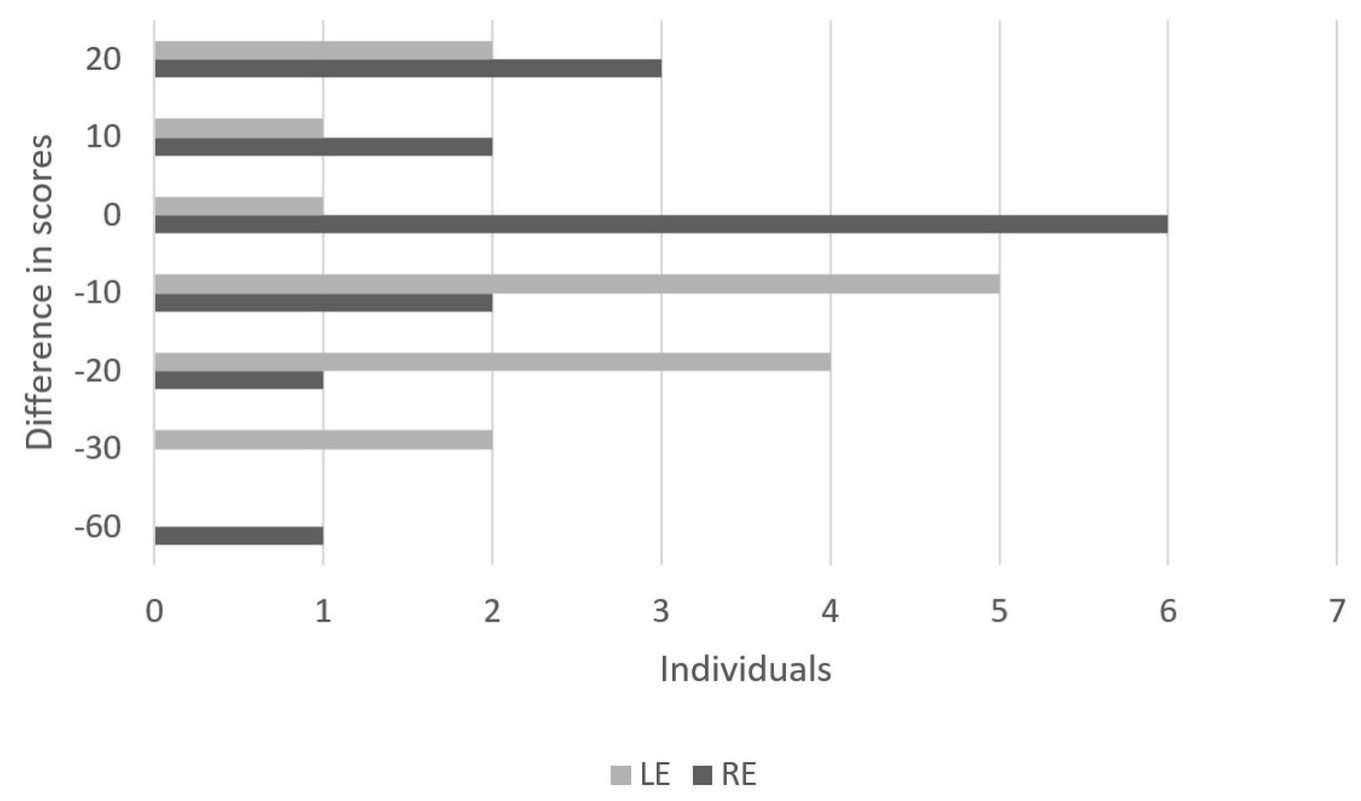
Difference between the scores obtained in the first and second application sequences in the divided attention step according to the ear side

**Figure 2:**
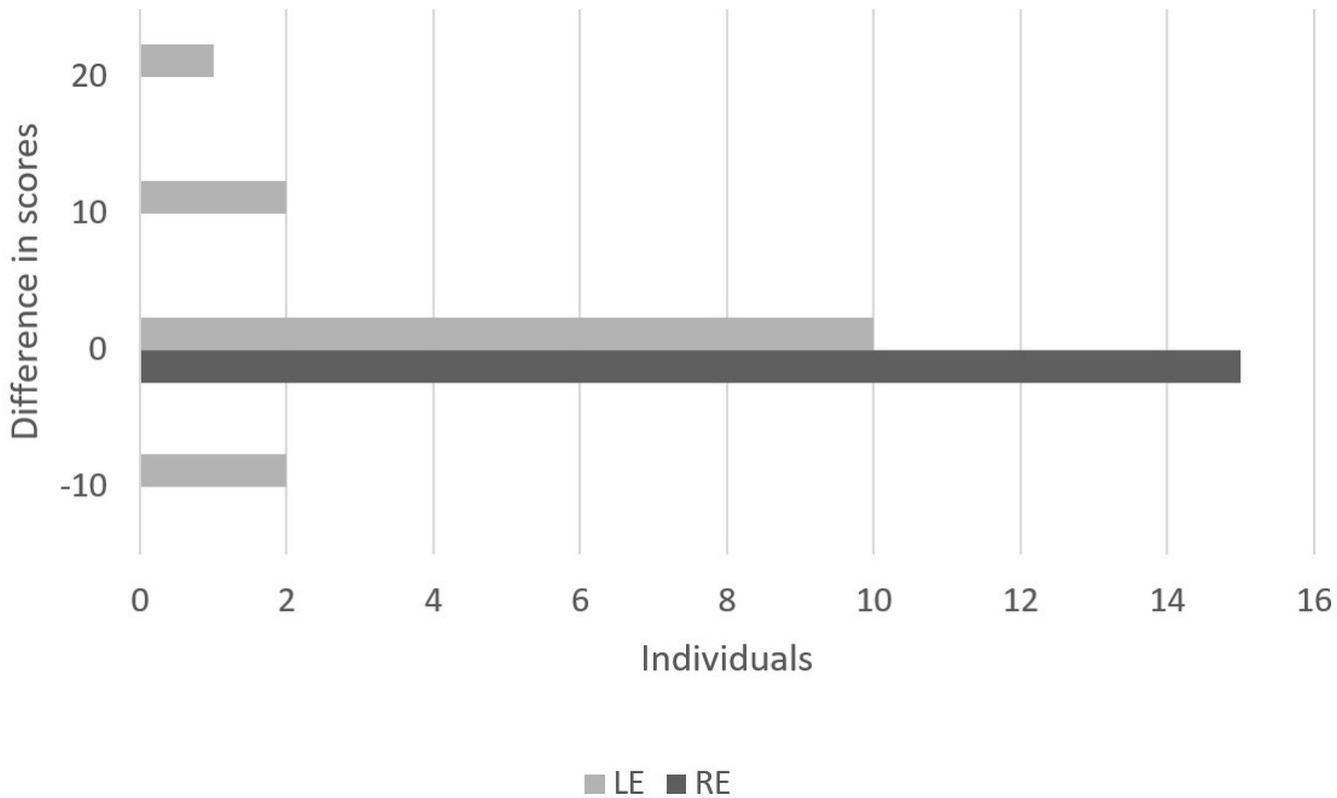
Difference between the scores obtained in the first and second application sequences in the directed attention step according to the ear side

In the comparison of the performance of adults with communicative complaints between Application Sequences 1 and 2 according to the ear side, one may observe the difference between the scores in the divided attention step in Figure 1 and the difference between the scores in the directed attention step in Figure 2.

**Caption:** LE = Left Ear; RE = Right Ear; negative scores: 1st sequence < 2nd sequence; 0: 1st sequence = 2nd sequence; positive scores: 1st sequence > 2nd sequence

**Caption:** LE = Left Ear; RE = Right Ear; negative scores: 1st sequence < 2nd sequence; 0: 1st sequence = 2nd sequence; positive scores: 1st sequence > 2nd sequence

## DISCUSSION

In the present study, based on the analysis of the performance of adults with communicative complaints evaluated with the SDT, in application sequence 1, we found that the mean performance in the divided attention stage was 84.67% in the right ear and 60.67% in the left ear (Table 1), which is lower than expected for this age group^(13)^. The mean value of correct answers found in a study carried out with the same test material^(13)^, which assessed adults without complaints, were 93.59% in the right ear and 86.06% in the left ear, and it was suggested, as normality by these authors in the RE, values greater or equal to 90% of correct answers and, in the LE, greater or equal to 80% of correct answers.

Results similar to these were found in a study carried out with the Dichotic Sentences Identification Test (DSI) in Brazilian Portuguese^(5)^, with an average performance of 93.70% of hits for the right ear and 88.60% for the left; despite having characteristics different from those of the test used in this study, it also showed similar results in adults with no complaints.

From these findings, it was possible to verify the influence of the communication complaints on the performance of normal-hearing adults assessed through the DST compared to other studies^(13,14)^ developed with normal-hearing adults with no complaints, considering that the complaints were the only diverging aspects between the analyzed groups.

The common behavioral manifestations reported during the initial interview, such as difficulty understanding speech in conflict situations (noise or speech) or reverberating acoustic environments, difficulty following fast speech, and frequent requests for repetition, among others, are possible indicators of risk of CAP alteration. Hence, these issues must be raised, considering that the basic audiological assessment employed may not indicate an alteration because it often ends up not representing the real functional impact since the CAP involves many processes measured at different levels of the central auditory system^(6)^.

Besides the inferior results relative to individuals with no complaints, this sample group presented a more considerable performance variation in the divided attention step, with results ranging from -50% (advantage of the left ear) to 60% (advantage of the right ear), whereas the differences varied from -20% to 30%for the group within normality^(13)^. During the literary search to compose this work, no studies were found with sample groups constituted of adults in the same age range as this study that could justify these findings, given that the heterogeneous profile and results so inferior to normality draw attention.

It was also found that the asymmetry between ears was significant, with a marked advantage of the right ear. The literature points to the existence of an advantage of the right ear in dichotic listening tests with right-handed individuals^(5-9)^ resulting from the left hemispheric dominance for linguistic stimuli^(10,15,16)^. Another aspect that justifies this advantage is related to the physiological structure - the dominant contralateral path, which presents more considerable complexity for the processing since the information received by the left ear is initially processed by the right hemisphere and transmitted via callous corpus to the left hemisphere, which in turn increases the acoustic processing time of the left ear, with there being a more significant demand for cognitive resources (attention, memory, executive function)^(8,17)^, so it is thus frequent and expected for there to occur a difference between the ears with the advantage of the right ear.

However, although the asymmetry is expected even for individuals deemed within normality, we highlight that this studied group presented an interaural asymmetry well above the expected for the age range^(13)^, with it being found that 80% of the individuals showed superior performance for the right ear, with score differences ranging from 10% to 60% between ears, and 20% presented a superior performance for the left ear, varying from -10% to -50% (Table 2), while another study referred as expected, within normality, a difference between ears up to 20%, with the advantage of the right ear^(12)^. The difference between ears in dichotic listening was also analyzed by researchers who assessed adult individuals with tone thresholds up to 40 dB HL and suggested that this difference cannot exceed 16%^(18)^.

The inferior performance and more considerable asymmetry between ears found here may be considered indicators of alterations to the auditory processing that may be related to the figure-ground skill, temporal processing, and auditory memory^(9,16,19)^.

Besides the relationship of the presented communication complaints with the auditory skills mentioned before, we must consider that attention and cognitive control are fundamental for the execution of dichotic listening, also influenced by aspects such as memory, language, and planning ability^(20-23)^. Factors such as memory and attention may act significantly on auditory perceptual asymmetry^(9)^.

In turn, in the directed attention step of the first application sequence, a performance equivalence was found, with hit scores varying from 90% to 100% in both ears (Table 1), which agrees with the findings of other studies carried out with the DST that presented slightly inferior results for the left ear relative to the right ear^(11,13,14)^.

Considering the different results of this sample group relative to the reference values for adults with no complaints present in the literature^(13)^, the analysis of the second application sequence was carried out to verify the constancy of the results, which allowed observing that, in the divided attention step, there was a tendency for better results for both ears, more pronounced for the left ear yet without statistical significance (Table 3). It should be noted that, despite the better performance presented with the second test protocol, it was observed that the performance variation and asymmetry between ears remained high relative to the performance shown in studies carried out with individuals in the same age range without communication complaints^(13,14)^. One may verify in the analysis of the difference between ears in the second application sequence that 13.3% of the individuals presented the same performance scores, 66.7% presented differences between ears ranging from 20% to 50% with better performance for the right ear, and 20% of the individuals showed a better performance for the left ear, with a difference between ears ranging from -30% to -20% (Table 4).

Another relevant aspect is the performance between the application sequences according to the ear side; it was found that, in the divided attention step, for the right ear, 40% of the individuals did not vary, 33% performed worse, and 26.7% performed better in the second application of the DST. In turn, there were more considerable variations between the results obtained in the different application sequences for the left ear, with only 6.6% of the individuals not varying, 20% performing worse, and 73.3% performing better (Figure 1).

As verified in the literature, the occurrence of improvements in the second application of an assessment is expected because it is related to the familiarization process^(23-25)^. However, although Application Sequences 1 and 2 were carried out the same day, it was possible to observe an improvement trend, yet the results obtained were remarkably heterogeneous, with considerable variability when compared to studies carried out with and without an interval between applications^(14,26)^. It was observed that, in many cases, when there was better performance for the worst ear (left), there was a decline for the best ear (right) in the divided attention step, which indicates that there was a change to the response strategy, thus demonstrating the difficulty that exists in this group for the more complex task.

In the directed attention task, there was no performance variation for the right ear, but the variation occurred for 33.33% of the individuals for the left ear, ranging from 20% to -10% between assessment protocols (Figure 2). A more homogeneous behavior was observed in this task, corroborating other research^(5,13)^.

Hence, based on the analysis performed and discussed here, the relevance of the communication complaints presented by the patient becomes evident, even if the basic audiological assessment and DDT results are within normality. With the difficulty level being higher for the DST due to the degree of cognitive aspect requirements, especially short-term memory and the high linguistic load level^(27)^, the difference in stimuli and complexity level between the DDT and DST may be a sign for us to direct our attention also to individuals who present communication complaints, even if the results of routine tests are within normality, given that such individuals perceive some difficulties in everyday communication, which may compromise their life quality and professional performance since most sound environments occur in unfavorable listening situations; therefore, the complaints must not be disregarded.

Moreover, it may be determined that the DST proved sensitive to indicate the possibility of auditory processing alterations, but its results must be analyzed together with other CAP tests and, when possible, with complementary assessments that help detect or exclude language or cognition alterations or other factors that may be influencing performance, such as the general health state of the patient, possible tiredness, concern, interest in the performance of the test, in addition to ensuring that the test instructions were understood correctly.

Thus, based on the joint analysis of these data, when an auditory processing alteration is confirmed, it is suggested that the individuals be referred to hearing rehabilitation.

## CONCLUSION

The performance of normal-hearing adults with communication complaints was inferior to that expected for the age range, with a striking difference between the ears, especially in the divided attention step, showing a heterogeneous profile due to the considerable response variability observed among the tested individuals and for the same individual when using two test protocols.
