# Supplementary material for: Dichotic Sentences Test Performance of Adults with communication complaints
Source: Codas. 2023 Aug 7;35(4):e20210301. doi: 10.1590/2317-1782/20232021301en (PMC10446753; doi:10.1590/2317-1782/20232021301en)
Supplement: Translation

# Translation: Desempenho no Teste Dicótico de Sentenças em Adultos com queixa comunicativa

**DOI:** 10.1590/2317-1782/20232021301pt

**Authors:** Geise Corrêa Ferreira, Eliane Schochat, Katya Marcondes Freire, Maristela Julio Costa

**Affiliations:** 1 Programa de Pós-graduação em Distúrbios da Comunicação Humana, Universidade Federal de Santa Maria - UFSM - Santa Maria (RS), Brasil.; 2 Departamento de Fisioterapia, Fonoaudiologia e Terapia Ocupacional, Faculdade de Medicina, Universidade de São Paulo - USP - São Paulo (SP), Brasil.; 3 Audicare - São Paulo (SP), Brasil.

**Keywords:** Audição, Percepção Auditiva, Testes Auditivos, Adulto, Anamnese

## Abstract

**Objetivo:**

Analisar o desempenho de adultos normo-ouvintes com queixa comunicativa no Teste Dicótico de Sentenças.

**Método:**

Selecionou-se em banco de dados, 15 participantes normo-ouvintes com resultados normais no Teste Dicótico de Dígitos, idades entre 19 a 44 anos, destros, que referiram queixa comunicativa. O Teste de Dicótico de Sentenças foi aplicado por meio de dois protocolos compostos por quatro diferentes combinações de listas denominados de sequências 1 e 2, de acordo com a seguinte ordem: treino, etapa de atenção dividida, etapas de atenção direcionada à direita e à esquerda.

**Resultados:**

Na sequência de aplicação 1, a média de desempenho na etapa de atenção dividida foi 84,67% na orelha direita e 60,67% na esquerda, com diferença estatística entre orelhas, com assimetria entre orelhas variando de -50% a 60%. Na etapa de atenção direcionada, a média de desempenho foi 99,33% na orelha direita e 98% na esquerda, sem diferença estatística. Na sequência de aplicação 2, houve tendência de melhores resultados, mais acentuada na orelha esquerda, sem significância estatística, mantendo elevadas a variação de desempenho e a assimetria entre orelhas. Verificou-se na comparação entre as sequências, na etapa de atenção dividida, que na orelha direita 40% dos indivíduos não variaram, 33% pioraram, 26,7% melhoraram; na orelha esquerda, 6,6% não variaram, 20% pioraram e 73,33% melhoraram. Na etapa de atenção direcionada houve estabilidade nos resultados.

**Conclusão:**

Os adultos normo-ouvintes, com queixas comunicativas, apresentaram um perfil heterogêneo, especialmente na etapa de atenção dividida, com acentuada diferença entre as orelhas e variabilidade de respostas.

## INTRODUÇÃO

O sentido da audição é o que nos torna capazes de apreender o mundo físico das vibrações sonoras e, a partir disto, formar as imagens mentais das experiências vivenciadas^(1)^. No nosso cotidiano, estamos expostos a inúmeros estímulos auditivos simultâneos, o que exige não somente a integridade da audição em nível periférico, mas como em nível central, para que uma comunicação efetiva se estabeleça^(2)^.

Na rotina clínica, há um aumento de indivíduos adultos que buscam avaliação audiológica por apresentarem baixo rendimento acadêmico ou profissional, relatando queixas como: dificuldades para ouvir, atenção, memória, mas principalmente de compreensão de fala, sobretudo, em situações desfavoráveis (com mensagem verbal competitiva). Entretanto, grande parte desses indivíduos, apresentam limiares auditivos dentro dos padrões de normalidade, o que por sua vez, torna fundamental a realização da avaliação do Processamento Auditivo Central (PAC), a fim de investigar a origem de tais queixas^(3)^.

O PAC corresponde a um conjunto de habilidades auditivas, responsáveis pela percepção e processamento das informações acústicas^(4)^. Para sua avaliação, há uma vasta gama de testes comportamentais que avaliam a função auditiva e sua relação com a comunicação^(5)^. Dentre eles estão os testes de escuta dicótica, sendo preconizado a utilização de pelo menos um destes testes na avaliação do PAC^(4,6)^.

Este tipo de tarefa pode ter como estímulo verbal: sílaba, dígitos, palavras e frases e correspondem a apresentação de dois estímulos competitivos concomitantes em ambas as orelhas, possibilitando a realização de tarefas de integração e separação binaural, com o objetivo de investigar a habilidade de figura-fundo^(7)^, bem como fornece informações sobre a atenção, maturação da via auditiva, função executiva, função hemisférica e inter-hemisférica, via corpo caloso^(8-10)^.

Recentemente, foi desenvolvido o Teste Dicótico de Sentenças (TDS)^(11)^, que visa investigar o desempenho do indivíduo em situações de escuta competitiva, por meio de estímulos compostos por frases simples que representam situações do cotidiano.

Por se tratar de um novo teste, com características diferentes dos demais testes de escuta dicótica encontrados na literatura nacional e internacional, são necessárias medidas para estabelecer parâmetros, em diferentes populações, que norteiem o avaliador na interpretação dos resultados, o que portanto justifica a realização desta pesquisa, que tem como foco investigar a sensibilidade do teste. Considerando os aspectos supracitados, o presente estudo teve como objetivo analisar e comparar o desempenho de adultos normo-ouvintes com queixa comunicativa no Teste Dicótico de Sentenças nas sequências de aplicação 1 e 2.

## MÉTODO

Trata-se de um estudo aplicado, de natureza experimental, quantitativo analítico de caráter retrospectivo realizado por meio da análise do banco de dados, vinculado a um projeto de pesquisa, previamente aprovado pelo Comitê de Ética em Pesquisa, sob o protocolo de nº 2.764.720. Para compor o grupo estudo, foram analisadas as avaliações de pacientes adultos normo-ouvintes com idade entre 19 e 44 anos realizadas entre o período de outubro de 2016 a fevereiro de 2017 que assinaram o Termo de Consentimento Livre e Esclarecido.

O grupo estudado foi selecionado a partir de um banco de dados composto por 101 indivíduos com idade entre 19 e 44 anos, os quais foram submetidos à anamnese específica, avaliação audiológica básica, para obtenção dos limiares tonais e avaliação das condições de orelha média. Já para avaliação das habilidades auditivas de integração e separação binaural, executou-se o teste Dicótico de dígitos na etapa de integração e o teste dicótico de sentenças, sequências 1 e 2, nas etapas de atenção dividida e direcionada à direita e à esquerda, cujos estímulos foram apresentados em um nível de 50 dB NS em relação à média tritonal (frequências de 500, 1000 e 2000 Hz).

Para a seleção da amostra foram considerados como critério de elegibilidade, indivíduos normo-ouvintes, considerando limiares de audibilidade valores iguais ou menores do que 25 dB NA^(12)^ e apresentar no Teste Dicótico de Dígitos (TDD), desempenho ≥ 95%^(1)^ para ambas as orelhas na etapa de integração binaural; preferência manual direita; ter no mínimo ensino médio completo; ter referido na anamnese uma ou mais queixas relacionadas à dificuldade de entender a fala em situações desfavoráveis de escuta, tais como em ambientes reverberantes ou na presença de ruído competitivo, bem como apresentar queixa de zumbido e aspectos relacionados à atenção e memória, salientando que estas foram apontadas pelos mesmos como fatores que pudessem estar associados à queixa principal referida.

Foram excluídos, aqueles que apresentaram excesso de cerume, possíveis aspectos condutivos, alterações neurológicas e/ou de fluência verbal evidentes ou autorrelatadas. Dessa forma, o grupo amostral foi composto por 15 indivíduos, sendo esses, seis do sexo masculino e nove feminino, com média etária de 28,7 anos.

As medidas com o Teste de Dicótico de Sentenças foram obtidas por meio da aplicação de dois protocolos compostos por quatro diferentes combinações de listas denominados de Sequências 1 e 2, de acordo com a seguinte ordem: treino, etapa de atenção dividida, etapa de atenção direcionada à direita e à esquerda^(11)^. Todos os participantes foram orientados sobre a forma de apresentação dos estímulos e a resposta (repetição verbal) solicitada para cada etapa das avaliações.

A sequência de aplicação 2 foi realizada com o objetivo de investigar a repetibilidade dos resultados obtidos na primeira aplicação, uma vez que trata-se de um novo instrumento, em que suas características tanto de estímulo verbal quanto de aplicação são atualmente diferentes de todos os testes dicóticos existentes na língua portuguesa e por esse motivo, não há um teste que possa ser considerado como padrão ouro para a análise de comparação e sensibilidade.

Quanto à análise de desempenho no TDS, a pontuação determinada foi de 10% para cada sentença, considerando que cada lista de aplicação de ambas as sequências são formadas por um conjunto de dez pares de sentenças, totalizando assim, 100% por orelha em cada etapa. Para analisar as respostas obtidas nas diferentes etapas do teste, foi considerada correta a repetição completa de toda a sentença apresentada^(11)^.

Para análise das diferenças dos escores obtidos entre orelhas, foi considerado que quando o escore foi negativo, significou que foi melhor na orelha esquerda; quando foi zero, que não houve diferença entre as orelhas, e quando foi positivo, que foi melhor na orelha direita.

Por sua vez, quando analisadas as diferenças dos escores obtidos entre as sequências 1 e 2, foram considerados de acordo com o lado da orelha, usando os seguintes critérios: quando obtido escore negativo, significou que o desempenho da respectiva orelha foi melhor na sequência 2 quando comparado com a sequência 1; quando foi zero, significou que em ambas as sequências foram obtidos os mesmos escores e, quando for positivo, significa que a sequência 1 teve escore melhor quando comparado à sequência 2.

As avaliações foram realizadas por uma única avaliadora, em uma sala acusticamente tratada, com o audiômetro Grason-Stadler modelo GSI-61, e fones auriculares modelo TDH 50. Os testes TDD e TDS, foram apresentados de forma gravada digitalmente em formato wav, por meio de um *tablet* acoplado ao audiômetro.

Os dados foram analisados estatísticamente, utilizando o programa SPSS. O teste Shapiro Wilk foi aplicado utilizando o nível de 5% como critério de determinação de significância para verificar a normalidade das variáveis. Para investigar o desempenho dos indivíduos no TDS e nas diferentes aplicações do instrumento, utilizou-se o teste não paramétrico de Wilcoxon, pois a hipótese de normalidade foi rejeitada. Foram consideradas significância estatística as análises que apresentaram p ≤ 0,05.

## RESULTADOS

Na Tabela 1 podem serem observados os valores absolutos de desempenho, nas etapas de atenção dividida e direcionada no TDS, de adultos com queixas comunicativas.

**Tabela 1:** Análise de desempenho no Teste Dicótico de Sentenças, nas etapas de atenção dividida e direcionada, na sequência de aplicação 1, de adultos normo-ouvintes com queixa comunicativa

	**ATENÇÃO DIVIDIDA**			**ATENÇÃO DIRECIONADA**		
	ORELHA DIREITA	ORELHA ESQUERDA	P-VALOR	ORELHA DIREITA	ORELHA ESQUERDA	P-VALOR
Média (DP)	84,67(16,85)	60,67(19,07)	0,020*	99,33(2,58)	98(4,14)	0,317
IQ	90(70-100)	60(50-60,0)		100(100-100)	100(100-100)	
MÍN- MÁX	40-100	30-100		90-100	90-100	

* Teste Wilcoxon, significância p≤ 0,05

**Legenda:** DP = Desvio Padrão; IQ = Intervalo Interquartílico; MÍN-MÁX = Mínimo-Máximo

Na Tabela 2, podem ser observadas as diferenças de desempenho obtidas entre as orelhas direitas e esquerdas na etapa de atenção dividida na sequência de aplicação 1.

**Tabela 2:** Diferença dos escores obtidos entre as orelhas direitas e esquerdas na sequência de aplicação 1, na etapa de atenção dividida, do Teste Dicótico de Sentenças (n=15)

Diferença (OD-OE)	Sequência de Aplicação 1 n(%)
-50	1(6,66)
-30	1(6,66)
-10	1(6,66)
0	0(0)
10	2(13,33)
30	1(6,66)
40	6(40,0)
50	2(13,33)
60	1(6,66)

**Legenda:** n = Número de Indivíduos; OD = Orelha Direita; OE = Orelha Esquerda

A comparação de desempenho dos adultos com queixa comunicativa nas etapas de atenção dividida e direcionada entre as sequências de aplicação 1 e 2 do TDS, podem ser observadas na Tabela 3.

**Tabela 3:** Comparação entre os desempenhos obtidos no Teste Dicótico de Sentenças, nas etapas de atenção dividida e direcionada, nas sequências de aplicação 1 e 2, em adultos com queixa comunicativa

	Sequência de Aplicação 1			Sequência de Aplicação 2			
ETAPAS	MÉDIA (DP)	IQ	MÍN-MÁX	MÉDIA (DP)	IQ	MIN-MÁX	P-VALOR
ATEN. DIVID.	84,67	90	40-100	86	90	60-100	0,809
OD	(16,85)	(70-100)		(13,52)	(70-100)		
ATEN. DIVID.	60,67	60	30-100	70	70	50-100	0,055
OE	(19,07)	(50-60)		(15,11)	(60-80)		
ATEN. DIREC	99,33	100	90-100	99,33	100	90-100	1,00
OD	(2,58)	(100-100)		(2,58)	(100-100)		
ATEN. DIREC.	98	100	90-100	96,67	100	80-100	0,480
OE	(4,14)	(100-100)		(6,17)	(90-100)		

Teste Wilcoxon

**Legenda:** DP = Desvio Padrão; IQ = Intervalo Interquartílico; MÍN-MÁX = Mínimo-Máximo; ATEN. DIVID = Atenção Dividida; ATEN. DIREC = Atenção Direcionada; OD = Orelha Direita; OE = Orelha Esquerda

Na Tabela 4 estão apresentadas as variações de desempenho entre as orelhas direitas e esquerdas obtidas com a aplicação da sequência 2 do TDS.

**Tabela 4:** Diferença dos escores obtidos entre as orelhas direitas e esquerdas, na sequência de aplicação 2, na etapa de atenção dividida, do Teste Dicótico de Sentenças (n=15)

Diferença (OD-OE )	Sequência de Aplicação 2 n(%)
-30	2(13,33)
-20	1(6,66)
0	2(13,33)
20	3(20,0)
30	3(20,0)
40	3(20,0)
50	1(6,66)

**Legenda:** n = Número de Indivíduos; OD = Orelha Direita; OE = Orelha Esquerda

Na comparação de desempenho dos adultos com queixa comunicativa entre as sequências de aplicação 1 e 2, segundo o lado da orelha, podem ser observadas na Figura 1 a diferença entre os escores na etapa de atenção dividida e na Figura 2 a diferença entre os escores na etapa de atenção direcionada.

**Figura 1:** Diferença entre os escores obtidos nas sequências de aplicação 1 e 2, na etapa de atenção dividida, segundo o lado da orelha

**Legenda:** OE = Orelha Esquerda; OD = Orelha Direita; escores negativos: sequência 1 < sequência 2; 0: sequência 1 = sequência 2; escores positivos: sequência 1 > sequência 2

**Figura 2:** Diferença entre os escores obtidos nas sequências de aplicação 1 e 2, na etapa de atenção direcionada, segundo o lado da orelha

**Legenda:** OE = Orelha Esquerda; OD = Orelha Direita; escores negativos: sequência 1 < sequência 2; 0: sequência 1 = sequência 2; escores positivos: sequência 1 > sequência 2

## DISCUSSÃO

No presente estudo, com base na análise do desempenho de adultos com queixas comunicativas, avaliados com o TDS, na sequência de aplicação 1, verificou-se que a média de desempenho na etapa de atenção dividida foi de 84,67% na orelha direita e de 60,67% na orelha esquerda (Tabela 1), desempenho esse, inferior ao esperado para a faixa etária^(13)^. As médias de acertos verificadas em estudo realizado com o mesmo material de teste^(13)^, que avaliou adultos sem queixa foram de 93,59% na orelha direita e 86,06%na orelha esquerda, tendo sido sugerido como normalidade por estes autores na OD valores maiores ou iguais a 90% de acertos e, na OE, maiores ou iguais a 80% de acertos.

Resultados semelhantes a estes, foram verificados em um estudo realizado com o teste de Identificação de Sentenças Dicóticas (DSI) em português brasileiro^(5)^, no qual apresentou um desempenho médio de 93,70% de acertos à direita e 88,60% à esquerda, que apesar de ter características diferentes do teste usado no presente estudo, também mostrou resultados semelhantes em adultos sem queixa.

A partir desses achados, foi possível verificar a influência da queixa comunicativa no desempenho de adultos normo-ouvintes, avaliados por meio do TDS, quando comparado com outros estudos^(13,14)^ desenvolvidos com adultos normo-ouvintes sem queixas, considerando ser a queixa o único aspecto divergente entre os grupos analisados.

As manifestações comportamentais comuns relatadas durante a entrevista inicial, como: dificuldade em compreender a fala em situações com conflito (ruído ou fala) ou em ambientes acústicos reverberantes, dificuldade em seguir uma fala rápida, solicitações frequentes de repetição, entre outras, são possíveis indicadores de risco para alteração do PAC^(6)^. Dessa forma, estas questões devem ser levadas em consideração mesmo que a avaliação audiológica básica utilizada, possa não indicar alteração, pois muitas vezes acabam não representando o real impacto funcional, considerando que o PAC envolve muitos processos, que são medidos em diferentes níveis do sistema auditivo central^(6)^.

Além dos resultados inferiores em relação aos indivíduos sem queixa, o presente grupo amostral apresentou maior variação de desempenho na etapa de atenção dividida, apresentando resultados que variaram de -50% (vantagem da orelha esquerda) a 60% (vantagem da orelha direita), enquanto para o grupo dentro da normalidade, as diferenças variaram de -20% a 30%^(13)^. Durante a busca literária para a composição deste trabalho, não foram encontrados estudos, cujo grupo amostral fosse constituído por adultos da mesma faixa etária do presente estudo, que pudesse justificar esses achados, uma vez que chama a atenção o perfil heterogêneo e resultados tão inferiores à normalidade.

Ainda foi verificado que a assimetria entre as orelhas foi significante, com vantagem acentuada da orelha direita. A literatura aponta a existência de vantagem da orelha direita em testes de escuta dicótica em indivíduos destros^(5,9)^ na qual é resultante da dominância hemisférica esquerda para estímulos linguísticos^(10,15,16)^. Outro aspecto que justifica essa vantagem, está relacionada com a estrutura fisiológica - com o caminho contralateral dominante, que apresenta maior complexidade para o processamento, uma vez que a informação recebida pela orelha esquerda é inicialmente processado pelo hemisfério direito e transmitida, via corpo caloso, para o hemisfério esquerdo, o que por sua vez, aumenta o tempo de processamento acústico da orelha esquerda, havendo maior demanda de recursos cognitivos (atenção, memória, função executiva)^(8,17)^, sendo portanto frequente e esperado que ocorra uma diferença entre as orelhas com vantagem da orelha direita.

Entretanto, apesar da assimetria ser esperada mesmo em indivíduos considerados dentro da normalidade, destacamos que este grupo estudado, apresentou um índice de assimetria interaural bem acima do esperado para a faixa etária^(13)^, na qual verificou-se que, 80% dos indivíduos apresentaram desempenho superior na orelha direita, com diferenças de escores de 10 a 60% entre as orelhas e 20% apresentaram desempenho superior na orelha esquerda, no qual variou de -10 a -50% (Tabela 2), enquanto outro estudo referiu como esperado, dentro da normalidade, uma diferença entre orelhas de até 20%, com vantagem de orelha direita^(12)^. A diferença entre orelhas na escuta dicótica foi analisada também por pesquisadores que avaliaram indivíduos adultos com limiares tonais de até 40dB NA, no qual sugerem que essa diferença não pode exceder 16%^(18)^.

O desempenho inferior e a maior assimetria entre orelhas verificados aqui, podem ser considerados como indicadores de alteração do processamento auditivo, que podem estar relacionadas à habilidade de figura-fundo, processamento temporal e memória auditiva^(9,16,19)^.

Além da relação das queixas comunicativas apresentadas, com as habilidades auditivas mencionadas anteriormente, devemos considerar que a atenção e o controle cognitivo, são fundamentais para a execução da escuta dicótica, sendo influenciada também por aspectos como memória, linguagem e capacidade de planejamento^(20-23)^. Os fatores como memória e atenção podem atuar de forma significativa na assimetria perceptual auditiva^(9)^.

Por sua vez, na etapa de atenção direcionada da sequência de aplicação 1, verificou-se equivalência de desempenho, cujo escores de acertos variaram de 90 a 100% em ambas as orelhas (Tabela 1), o que vai ao encontro também, dos achados de outros estudos realizados com o TDS, no qual apresentaram resultados levemente inferiores na orelha esquerda em relação à orelha direita^(11,13,14)^.

Considerando os resultados distintos do presente grupo amostral, em relação aos valores de referência de adultos sem queixas, presentes na literatura^(13)^, foi realizada a análise da sequência de aplicação 2, a fim de verificar a constância dos resultados, o que permitiu observar que na etapa de atenção dividida houve uma tendência de melhores resultados, em ambas as orelhas, mais acentuada na OE, mas sem significância estatística (Tabela 3). Destaca-se que apesar do melhor desempenho apresentado com a apresentação do segundo protocolo de teste, foi observado que a variação de desempenho e a assimetria entre as orelhas se mantiveram elevadas em relação ao desempenho apresentados em pesquisas realizadas com indivíduos de mesma faixa etária sem queixas comunicativas^(13,14)^. Pode ser verificada na análise da diferença entre as orelhas, na sequência de aplicação 2, que 13,3% dos indivíduos apresentaram os mesmos escores de desempenho, 66,7% apresentaram diferença que variou de 20 a 50% entre as orelhas, com melhor desempenho na orelha direita e 20% dos indivíduos apresentaram desempenho melhor na orelha esquerda, com diferença entre orelhas de -30 a -20% (Tabela 4).

Outro aspecto relevante é o desempenho entre as sequências de aplicação, de acordo com o lado da orelha, no qual verificou-se que na etapa de atenção dividida, na orelha direita 40% dos indivíduos não variaram, 33% pioraram, e 26,7% melhoraram na segunda sequência de aplicação do TDS. Por sua vez, a orelha esquerda, apresentou maior variação entre os resultados obtidos nas diferentes sequências de aplicação, sendo que apenas 6,6% não variaram, 20% pioraram e 73,33% melhoraram (Figura 1).

Conforme verificado na literatura, a ocorrência de melhora na segunda aplicação de uma avaliação é esperada, pois está relacionada com o processo de familiarização^(23-25)^. Entretanto, apesar das sequências de aplicações 1 e 2 terem sidas realizadas no mesmo dia, foi possível observar uma tendência de melhora, porém os resultados obtidos foram muito heterogêneos, com uma grande variabilidade quando comparados com estudos realizados com e sem um intervalo de tempo entre as aplicações^(14,26)^. Observou-se que em muitos casos, quando houve melhor desempenho na pior orelha (esquerda), ocorreu um rebaixamento da melhor orelha (direita), na etapa de atenção dividida, o que indica que houve mudança na estratégia de resposta, demonstrando assim, a dificuldade existente nesse grupo para a tarefa mais complexa.

Na tarefa de atenção direcionada, na orelha direita não houve variação de desempenho e, na orelha esquerda, a variação ocorreu em 33,33% dos indivíduos, cuja faixa foi de 20 a -10% entre os protocolos de avaliação (Figura 2). Nessa tarefa foi observado um comportamento mais homogêneo, corroborando outras pesquisas^(5,13)^.

Assim sendo, com base nas análises aqui realizadas e discutidas, fica evidente a relevância das queixas comunicativas apresentadas pelo paciente, mesmo que os resultados da avaliação audiológica básica e do TDD estejam dentro da normalidade. A diferença de estímulos e nível de complexidade entre TDD e TDS, cujo grau de dificuldade é maior no TDS, decorrente do nível de exigência de aspectos cognitivos, em especial memória de curto prazo e do alto nível de carga linguística^(27)^, pode ser um sinalizador para que voltemos nossa atenção também para indivíduos desta faixa etária que apresentam queixas comunicativas, mesmo que os resultados dos testes de rotina estejam dentro da normalidade, pois estes indivíduos percebem algumas dificuldades na comunicação do dia -a -dia, o que pode comprometer a sua qualidade de vida e desempenho profissional, uma vez que a maioria dos ambientes sonoros ocorrem em situações desfavoráveis de escuta, e portanto as queixas não devem ser desconsideradas.

Ainda, pode ser constatado que o TDS se mostrou sensível para indicar possibilidade de alteração do processamento auditivo, mas seus resultados devem ser analisados em conjunto com outros testes de PAC e quando possível, com avaliações complementares que auxiliem a detectar ou excluir alteração de linguagem e cognição, ou outro fator que possa estar influenciando o desempenho, como o estado geral de saúde do paciente, possível cansaço, preocupação, interesse na realização do teste, assim como garantir que as instruções do teste tenham sido entendidas adequadamente.

Assim, com base na análise conjunta destes dados, quando confirmada alteração do processamento auditivo, sugere-se que os indivíduos sejam encaminhados para reabilitação auditiva.

## CONCLUSÃO

O desempenho de adultos normo-ouvintes com queixas comunicativas foi inferior ao esperado para a faixa etária, com uma acentuada diferença entre as orelhas, especialmente na etapa de atenção dividida, mostrando perfil heterogêneo, devido à grande variabilidade de respostas observada, tanto entre os indivíduos testados, como do mesmo indivíduo, ao usar dois protocolos de teste.
